# A comparative study of meat quality and vibrational spectroscopic properties of different chicken breeds

**DOI:** 10.1016/j.psj.2022.101829

**Published:** 2022-03-08

**Authors:** Sasikan Katemala, Amonrat Molee, Kanjana Thumanu, Jirawat Yongsawatdigul

**Affiliations:** ⁎ Shcool of Food Technology, Institute of Agricultural Technology, Suranaree University of Technology, Nakhon Ratchasima, 30000, Thailand; † School of Animal Technology and Innovation, Institute of Agricultural Technology, Suranaree University of Technology, Nakhon Ratchasima, 30000, Thailand; ‡ Synchrotron Light Research Institute (Public Organization), Nakhon Ratchasima, 30000, Thailand

**Keywords:** chicken breed, meat quality, synchrotron radiation-based Fourier transform infrared microspectroscopy, Fourier transform Raman spectroscopy, principle component analysis

## Abstract

Chicken breed is one of the key factors that influence meat quality. The quality attributes of breast meat from commercial broiler (**CB**), Thai native chicken (**NC**, Leung Hang Khao), and the crossbred Korat chicken (**KC**) were investigated via synchrotron radiation-based Fourier transform infrared (**SR-FTIR**) microspectroscopy, Fourier transform Raman (**FT-Raman**) spectroscopy, and physicochemical analysis. The protein and carbonyl contents of KC and NC meats were higher than that of CB meat, but the lipid content was lower (*P* < 0.05). CB meat was characterized by high moisture, lightness (L*), and presence of taste-active nucleotides, namely, inosine 5′-monophosphate (**IMP**) and guanosine 5′-monophosphate (**GMP**). Moreover, NC meat had the highest insoluble collagen and inosine contents (*P* < 0.05). The predominant protein secondary structures of KC and NC meats were β-turns and random coils, whereas α-helices were mainly found in CB meat. Based on principal component analysis, the meat quality and spectra were clearly separated by breeds. The high moisture and lipid content of meat corresponded to O–H stretching (3,203 cm^−1^) and C–H stretching (2,854 cm^−1^) in the FT-Raman spectra, whereas PO_2_^−^ stretching (1,240 cm^−1^), measured via SR-FTIR, was well correlated with the IMP content. In addition, the FT-Raman wavenumber of 934 cm^−1^, indicating C–C stretching, was correlated with high water-holding capacity (**WHC**) in KC meat. The quality of meat from slow- and fast-growing chickens significantly varies. Vibrational spectroscopy is a powerful technique that provides insightful molecular information correlated with various meat attributes.

## INTRODUCTION

The consumption of chicken meat has increased worldwide owing to its emerging reputation as a healthy white meat with low fat and high protein content. Fast-growing broiler strains, with a rearing period of 5 to 6 wk, are mainly used to produce commercial chicken meat (Choe et al., 2010). Native chicken (**NC**) meat has gained consumer acceptance, particularly in Asian countries, owing to its unique taste and texture, as well as its perception as healthier than commercial broiler (**CB**) (Jaturasitha et al., 2008; Jayasena et al., 2014). NC generally has a slower growth rate than CB with the rearing period of 16 to 20 wk. Thus, NC meat has not been produced in sufficient numbers to meet consumer demand because of its slow growth rate, poor feed efficiency, and low lean-muscle-gaining ability (Choe et al., 2010). Consequently, crossbred chickens have been developed to genetically improve production capacity (Khawaja et al., 2013; Maliwan et al., 2017; Wang et al., 2019). The quality of meat significantly varies depending on chicken breed. Color is the major characteristic and indicator of meat quality. The variation in color among poultry breeds is influenced by varying amounts of myoglobin and pH (Froning, 1995; Fletcher, 1999). The taste and texture attributes contribute to the perception of meat delicacy. NC meat has unique taste and texture characteristics. It has been reported that the umami taste of poultry meat arises mainly from taste-active nucleotides, predominantly inosine 5′-monophosphate (**IMP**), which was notably higher in NCs than in CBs (Fujimura et al., 1994; Ahn and Park, 2002). Tenderness is the most important aspect of meat texture and is related to the proportion of crosslinked collagen. In general, the older slow-growing chickens have a higher degree of collagen crosslinking, which results in less-tender meat (Wattanachant et al., 2004). The water-holding capacity (**WHC**) also affects the appearance and texture variables of meat. However, which chicken breed has the best WHC is still unknown (Choe et al., 2010; Katemala et al., 2021). With the increasing consumer preference for healthier products, the nutritional compositions of meat, such as protein and fat, have a crucial influence on its quality. At present, protein oxidation in meat has gained interest owing to its influence on meat quality and human nutrition. Protein oxidation impairs quality traits, including tenderness, WHC, juiciness, and susceptibility to proteolysis (Estévez, 2011; Lund et al., 2011). The extent of protein oxidation is affected by reactive oxygen species (**ROS**) during meat maturation, which is likely to vary by breed. Leung Hang Khao is an indigenous chicken breed in Thailand, and the available scientific information on its meat quality is limited. Korat chicken (**KC**) is a crossbreed between Leung Hang Khao sires and SUT 101 chicken dams (a crossbreed between broiler and layer chickens) and was developed by the Suranaree University of Technology (**SUT**); it exhibits improved growth performance compared with NC (Maliwan et al., 2017). However, the meat quality of KC has not been systematically investigated. A comparison of the meat quality of Leung Hang Khao and Korat chickens with that of CB would allow the valorization of native and crossbred chickens.

Vibrational spectroscopies, including Fourier transform infrared (**FTIR**) and Fourier transform Raman (**FT-Raman**), have emerged as rapid and nondestructive methods for characterizing meat quality in relation to its structural compositions, including proteins, water, lipids, and carbohydrates (Ellis et al., 2005; Boyaci et al., 2014; Deniz et al., 2018; Sinanoglou et al., 2018). The benefits of this technique are the direct contact with meat samples and the provision of information on the structural conformational changes at the molecular level within intact cells without sample pre-treatment. The IR spectra show the transmitted, reflected, or dispersed radiation originating from the changes in molecular dipoles associated with vibrations and rotations. The Raman spectra originate from the inelastic scattering of the incident light and depend on the changes in the polarizability of functional groups when atoms vibrate (Li-Chan, 1996; Hashimoto et al., 2019). Therefore, IR and Raman spectroscopies are complementary techniques that provide molecular information on polar and non-polar groups, respectively. Synchrotron radiation-based Fourier transform infrared (**SR-FTIR**) spectroscopy is another advanced technique that provides higher spatial resolution with low signal-to-noise ratio compared with a benchtop-source (Globar) FTIR (Miller and Dumas, 2006). Synchrotron radiation (**SR**) is an electromagnetic radiation emitted from a photo or electron accelerated to near the speed of light. Its advantages are high brightness, extreme intensity, and beam focus of 10 to 20 μm, all of which allow the characterization of the microstructures of biological tissues. The use of SR-FTIR in the measurement of meat quality is still rare; however, it is expected that in-depth molecular information on chicken meat muscle can be obtained from SR-FTIR analysis.

This study aimed to compare the meat quality of crossbred KC and Thai NC (Leung Hang Khao) with that of CB at their respective market ages by assessing their physicochemical properties and collecting SR-FTIR and FT-Raman spectra. The correlations between spectroscopic data and meat quality were established using principle component analysis (**PCA**) to identify the relationships between the spectra features and meat quality traits of different breeds.

## MATERIALS AND METHODS

### Animal and Sample Preparation

All the procedures employed in the present study were approved by the Animal Care and Use Committee of SUT, Thailand. In total, 120 one-day-old mixed-sex KC and NC were randomly distributed to three pens (40 chicks/pen/5 m^2^) in an indoor facility at the SUT Farm (Nakhon Ratchasima, Thailand) and raised under the same conditions, yielding three replicates of each breed. The birds were fed ad libitum with the same commercial diet at the starter (0 to 3 wk old), grower (4 to 6 wk old), and finisher (7 to slaughter) stages, containing 21%, 19%, and 17% of crude protein, respectively. The birds had free access to water and no access to the outdoor environment. When the birds reached the market age (10 wk for KC and 16 wk for NC), 36 male chicks (12 chicks/pen) of each breed were randomly selected and fasted for 12 to 15 h, weighed (KC: 1.40–1.82 kg; NC: 1.40–2.04 kg), and then slaughtered in a commercial slaughterhouse (Nakhon Ratchasima, Thailand). The birds were processed in accordance with the commercial standards: first was stunning by electrocution, followed by conventional neck cutting, bleeding, scalding, plucking, and eviscerating. The carcasses were then placed in an ice box and then transferred to the laboratory within 1 h. The breast meat samples were collected after storage for 24 h in a 4°C chiller. The skin, bone, visible connective tissue, and fat were removed. The breast meat samples of male CB at 6-wk-old with a live weight of 2.9 to 3.0 kg were obtained from a commercial chicken meat processing company (Charoen Pokphand Foods Public Company Limited, Nakhon Ratchasima, Thailand) and stored for 24 h in a 4°C chiller. The pH at 24 h postmortem was measured, and the WHC was measured within 24 h. The color of the meat was determined within 48 h. The samples were also allocated for SR-FTIR spectroscopic measurement. The remaining samples were minced, vacuum-packed, and stored at −80°C for Raman spectroscopy and other analyses within 1 month. The frozen samples were thawed in a refrigerator at 4°C for 12 to 18 h before analysis.

### Proximate Composition and Physicochemical Properties

The moisture content, crude protein, and ash were determined using the AOAC (2010) method. The chloroform–methanol extraction method described by Folch et al. (1957) was employed to determine the total lipid content. At 24 h postmortem, the pH values were measured in accordance with the method described by Wattanachant et al. (2004). Before the pH measurement (MP220; Mettler-Toledo, Schwerzenbach, Switzerland), approximately 1 g of mince was homogenized in 5 mL of distilled water for 30 s using a homogenizer (Ultra Turrax T25; Ika Werke GmbH & Co., Staufen, Germany).

The color of the breast meat was measured using a colorimeter (Hunter Associates Laboratory, Reston, VA), which was standardized using a light trap (black hole) and white tiles. The color values were recorded in accordance with the Commission Internationale de l'Eclairage (**CIE**), namely, lightness (L*), redness (a*), and yellowness (b*), using a light source of D65 (daylight, 65° light angle) and measured at three different locations on the meat surface.

The WHC was measured in accordance with the method described by Ryoichi et al. (1993), with some modifications. Briefly, 2 g of breast meat was placed on filter paper (No. 4; Whatman International Ltd., Maidstone, UK) and centrifuged at 6,710 × *g* for 10 min at 25°C. The WHC was expressed as percentage of the weight absorbed by the filter paper to the moisture content of the original meat sample.

### Nucleotides

The nucleotide content of the meat samples was measured in accordance with the method described by Kim et al. (2012), with slight modifications. The samples (5 g) were homogenized with 50 mL of 7.5% cold perchloric acid (Ultra Turrax T25; Ika, Werke GmbH & Co., Staufen, Germany), centrifuged at 2,000 × *g* for 5 min at 4°C, and the supernatant was collected. The extract was then mixed with 0.6 M of neutralizing buffer (pH 7.6; KH_2_PO_4_ + K_2_HPO_4_). After 10 min, the supernatant was filtered through a 0.45-µm nylon filter and analyzed via HPLC (HP 1260; Agilent Technologies, Inc., Santa Clara, CA) equipped with a C_18_ reverse-phase column (Hypersil ODS, 4.6 × 150 mm, 3-μm particles) (Thermo Scientific, Waltham, MA). The injection volume was 10 μL, and the elution time was 25 min; the mobile phases were A (150-mM KH_2_PO_4_ and 150-mM KCl, pH 6) and B (mobile phase A mixed with 20% acetonitrile) at a flow rate of 0.5 mL/min. The composition of the mobile phase was 3% B for 0 to 5 min; increased to 9% B for 5 to 10 min, 20% B for 10 to 15 min, and 100% B for 15 to 20 min; and then maintained at 100% B for 5 min. The column temperature was 25°C, and the detection wavelength was 254 nm. The quantities of IMP, guanosine 5′-monophosphate (**GMP**), adenosine triphosphate (**ATP**), adenosine diphosphate (**ADP**), adenosine monophosphate (**AMP**), inosine, and hypoxanthine were calculated with reference to the external standards (Sigma-Aldrich Co., St. Louis, MO).

### Collagen Content

The total collagen content was determined via alkaline hydrolysis, as described by Reddy and Enwemeka (1996). The samples were hydrolyzed with 7M sodium hydroxide (NaOH) at 120°C for 40 min. The hydrolysate was neutralized with 3.5M sulfuric acid (H_2_SO_4_), filtered, and reacted with chloramine T solution and Ehrlich's reagent. The absorbance at 550 nm was measured using a spectrophotometer (Jenway, Bibby Scientific Ltd., Stanffordshore, UK). The amount of hydroxyproline was determined, and the total collagen content was calculated using a factor of 7.25 (Bergman and Loxley, 1963).

The content of insoluble collagen was determined in accordance with the method described by Liu et al. (1996). The meat samples were homogenized with 25% Ringer's solution, and the homogenates were heated to 77°C for 70 min in a water bath and then centrifuged at 2,300 × *g* for 30 min at 4°C. The extraction was repeated twice, and the residues were dried overnight at 105°C. The insoluble collagen content of the residues was determined and calculated as described above.

### Protein Carbonyl Content

Protein oxidation was measured from the total carbonyl content and evaluated using 2, 4-dinitrophenylhydrazine (**DNPH**), as described by Mercier et al. (2004), with some modifications. The chicken breast samples were minced and homogenized in 20 mM sodium phosphate buffer (pH 6.5) in a ratio of meat to buffer of 1:10 (wt/vol) using a homogenizer (Ultra Turrax T25, Ika, Werke GmbH & Co., Staufen, Germany) for 30 s. After the addition of 0.5 mL of 20% trichloroacetic acid (**TCA**), the precipitate was collected and mixed with 0.2% DNPH to form 2,4-dinitrophenylhydrazones and washed with 5 mL of a 1:1 (vol/vol) mixture of ethanol and ethyl acetate until a clear supernatant was obtained. The mixture was dried under N_2_ gas and dissolved in 2 mL of 20 mM sodium phosphate buffer (pH 6.5) containing 6 mM guanidine hydrochloride. The absorbance at 370 nm was measured using a spectrophotometer (Jenway, Bibby Scientific Ltd., Stanffordshore, UK) and expressed as nM of carbonyl per mg protein using an extinction coefficient of protein hydrazones of 21.0 mM^−1^ cm^−1^. The protein concentration of the control sample (without the addition of DNPH) was measured using the Bradford assay. A standard curve of bovine serum albumin was constructed at concentrations ranging from 0 to 1.0 mg/mL.

### Vibrational Spectroscopic Measurement

#### Synchrotron Radiation-Based Fourier Transform Infrared (SR-FTIR) Microspectroscopy

The spectra were collected at room temperature using an FTIR spectrometer (Hyperion 2000; Bruker Optics, Ettlingen, Germany) coupled with an infrared microscopy 15 × objective lens, equipped with a MCT D315 detector cooled with liquid nitrogen at an infrared microspectroscopy beamline (BL4.1 Infrared Spectroscopy and Imaging). SR was obtained from the BM4 of the 1.2-GeV storage ring at the Synchrotron Light Research Institute (Public Organization) (Nakhon Ratchasima, Thailand) and used as the infrared radiation source. The samples were embedded in optimal cutting temperature compound, snap-frozen in liquid N_2_, and stored at −80°C until preparation into 6-µm sections using a cryomicrotome (Microm HM525; Thermo Fisher Scientific, Walldorf, Germany). Specimens were placed on barium fluoride (BaF_2_) windows and dried in a vacuum chamber overnight before SR-FTIR analysis in the transmission mode. Spectra were acquired from the measurement area of 200 × 200 µm^2^ over the wavenumber range of 4,000 to 800 cm^−1^ using an aperture size of 10 × 10 µm^2^ with a spectral resolution of 4 cm^−1^ and 64 scans. At least 30 spectra were collected from each sample and averaged to represent one sample. Three replications of independent lots were carried out in each chicken breed, resulting in a total of at least 90 spectra per chicken breed. The spectrometer was purged with N_2_ and a background spectrum of BaF_2_ windows was recorded to reduce spectral contributions from water vapor. The baseline was estimated using the automated background removal. IR spectra were also pre-processed by water compensation method in the OPUS 7.2 software (Bruker Optics Ltd., Ettlingen, Germany).

#### Fourier Transform Raman (FT-Raman) Spectroscopy

The thawed minced samples were equilibrated to room temperature prior to Raman spectroscopy measurements. The Raman spectra were collected on a Bruker Vertex 70 FT-Raman spectrometer (Bruker, Karlsruhe, Germany) over the wavenumber range of 4,000 to 400 cm^−1^ at a spectral resolution of 4 cm^−1^ and 256 scans. Sulfur was used to calibrate the Raman frequency. A diode-pumped Nd/YAG laser at 1,064 nm with an output of 500 mW of laser power was used as the excitation source. FT-Raman spectral acquisition and instrument control were performed using the OPUS 7.2 software (Bruker Optics Ltd., Ettlingen, Germany). Ten spectra of individual samples were collected and averaged to represent one sample. Three replications of independent lots were performed with at least 30 spectra for each chicken breed.

#### Spectra Processing and PCA Analysis

**Figure 1:**
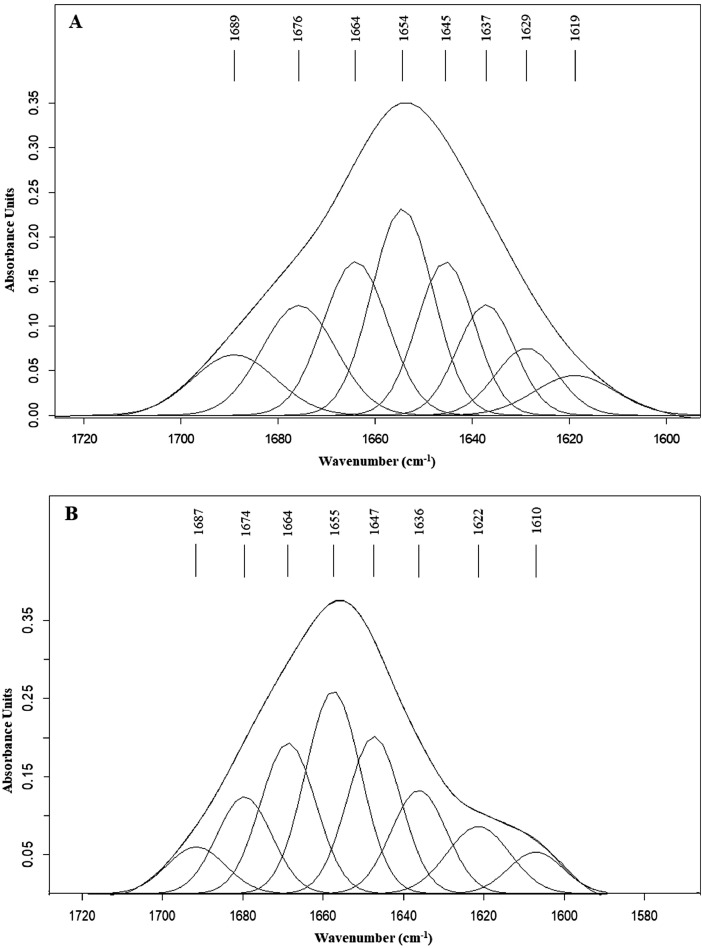
The amide I band of SR-FTIR (A) and FT-Raman (B) spectra after contouring with best-fit 50% Loentzian/Gaussian individual component bands.

To further evaluate changes in the secondary structure, the IR and Raman spectra were preprocessed via smoothing by applying a 13-points, vector normalization against the amide I region (1,700–1,600 cm^−1^), and baseline scattering correction to enhance the resolution of superimposed bands and to minimize problems from unavoidable baseline shifts. Preprocessed spectra were curve-fitted in the 1,700 to 1,600 cm^−1^ region using appropriate Gaussian and Lorentzian functions in the OPUS 7.2 software (Bruker Optics Ltd., Ettlingen, Germany). The curve fit of amide I band provides the estimate of the secondary structure by integration of band area as shown in Figure 1. Protein secondary structure including α-helix, β-sheet, random coil, β-turn is shown in Table 3. The PCA were analyzed using Unscrambler X 10.5.1 (Camo Analytics, Oslo, Norway). Data were processed using the Savitzky-Golay algorithm (with 9 and 13 smoothing points for IR and Raman spectra, respectively), baseline correction, and then normalized using Extended Multiplicative Signal Correction using the IR spectra in the ranges of 3,801 to 2,704, 1,802 to 899 cm^−1^ and of the Raman wavenumbers of 3,801 to 2,704, 1,803 to 399 cm^−1^. Seven principles components (**PCs**) were chosen for analysis. The wavenumbers with a high loading were selected from loading plots. Subsequently, the selected wavenumbers, along with data of secondary structure, and physicochemical properties for the PCA analysis were determined using Unscrambler X 10.5.1 (Camo Analytics, Oslo, Norway). The weighting method used for the PCA analysis was 1/standard deviation (**SD**) when investigating the relationships with other variables.

### Statistical Analysis

Comparison of the quality of CB, KC, and NC meat was performed using a completely random design. All analytical experiments were conducted in triplicate. A one-way ANOVA was employed to analyze the effects of chicken breed on meat quality. Comparisons of the means were performed using Tukey's test. Statistical significance was accepted for *P* < 0.05. All statistical analyses were conducted using SPSS 16.0 (SPSS Inc., Chicago, IL) to compare the means derived from each chicken breed within each analysis, and the results were expressed as mean ± SD.

## RESULTS AND DISCUSSION

### Proximate Composition and Physicochemical Properties

The protein contents of KC and NC meats were higher than that of CB meat, but the moisture and total lipid contents were lower (*P* < 0.05; Table 1). The higher protein content of native and crossbred chicken meats compared with that of broiler meat has been reported in Korean, Japanese, and Thai chickens (Wattanachant et al., 2004; Rikimaru and Takahashi, 2010; Jung et al., 2014). The differing protein contents of chicken meat may be due to the combination of breed and age. A higher fat content was observed in broiler meat than in the meat of older slow-growing birds (Wattanachant et al., 2004; Rikimaru and Takahashi, 2010; Ismail and Joo, 2017). The meats of fast-growing chickens with good muscle development and growth had a higher intramuscular fat content than the meats of slow-growing chickens (Ismail and Joo, 2017). Lipid biosynthesis appeared to occur to a greater extent in CBs, whereas local chicken breeds had higher levels of lipid degradation (Zheng et al., 2016). Such physiological differences may explain the higher lipid contents in broiler chicken meat. The ash content of KC meat was lower than that of CB and NC meats (*P* < 0.05); this finding was consistent with that of a previous study (Wattanachant et al., 2004). With regard to meat color, the lightness (L*) value was lower in KC meat than in CB meat (*P* < 0.05), whereas the redness (a*) and yellowness (b*) values were comparable among all the three breeds (*P* > 0.05). KC meat was darker than CB meat (*P* < 0.05), which was likely due to the higher concentration of myoglobin and heme pigments (Wideman et al., 2016). The pH of KC and NC meats was lower than that of CB meat. It has been previously reported that the meat of fast-growing birds has a higher the ultimate pH value than that of slow-growing birds (Wattanachant et al., 2004; Berri et al., 2005). The meat of fast-growing birds had a lower glycogen content than that of slow-growing birds, leading to the limited conversion of glycogen to lactic acid after death (Berri et al., 2005; Talpur et al., 2018). Moreover, slow-growing chickens struggled more during shackling, which resulted in faster muscle acidification after death compared with fast-growing broilers (Berri et al., 2005). WHC is a complex trait that depends on the composition and structure of the muscle tissues. KC meat had the highest WHC (*P* < 0.05). Our studies demonstrated that the meat quality significantly varied depending on the breed.

**Table 1:** Proximate composition and physico-chemical properties of breast meat of three chicken breeds (mean ± SD).

Parameters	CB	KC	NC
Moisture (%)	76.98 ± 0.45^a^	75.22 ± 0.27^b^	75.10 ± 0.34^b^
Crude protein (%, db)	85.02 ± 3.42^b^	95.61 ± 0.85^a^	96.21 ± 0.91^a^
Total lipid (%, db)	6.08 ± 0.71^a^	3.32 ± 0.08^b^	3.22 ± 0.09^b^
Ash (%, db)	4.93 ± 0.05^a^	4.49 ± 0.06^b^	5.00 ± 0.06^a^
pH	5.92 ± 0.19^a^	5.65 ± 0.04^b^	5.83 ± 0.22^ab^
WHC (%)	61.35 ± 4.77^b^	66.80 ± 2.62^a^	59.50 ± 2.35^b^
L*	63.71 ± 4.64^a^	57.53 ± 4.69^b^	60.81 ± 7.34^ab^
a*	2.56 ± 2.38	2.39 ± 3.11	3.30 ± 2.31
b*	10.90 ± 5.32	11.72 ± 5.89	12.70 ± 4.16

Abbreviations: CB: commercial broiler; KC: Korat hybrid chicken; NC: Thai native chicken; WHC: water holding capacity; db: dry basis.

a-b Mean values within a row with different superscripts differ significantly (*P* < 0.05).

### Nucleotides

IMP was a major nucleotide in the breast meat from all 3 breeds. CB meat contained the highest IMP and GMP contents, whereas NC meat contained the lowest amount (*P* < 0.05; Table 2). The variation in the IMP concentration was primarily related to muscle development processes and regulatory genes (Ma et al., 2015). Compared with ADP and AMP, the content of ATP was relatively low in all 3 breeds. The low ATP content indicated that ATP was almost completely depleted after slaughter and was degraded to ADP, AMP, and other derivatives. The rate of ATP decrease in KC and NC meat appeared to be higher than that in CB meat Vani et al. (2006). reported that the degradation rate of IMP was higher at acidic pH than at neutral or alkaline pH. NC meat contained the highest inosine content. The variation in fiber composition among chicken breeds determined the inosine content. Native chickens contain more oxidative fibers (type I fibers) than domesticated birds (Jaturasitha et al., 2017) Tullson and Terjung (1999). reported that the activity of 5′-nucleotidase, which catalyzed the degradation of IMP to inosine, was higher in type I muscle fiber than in type II muscle fiber in rat skeletal muscles. These intrinsic factors may partly explain the variations in these nucleotides among breeds. Hypoxanthine was not detected in any breed.

**Table 2:** Chemical parameters related to meat quality of chicken breast meat from three different breeds (mean ± SD).

Parameters	CB	KC	NC
Nucleotides (µg/g, db)			
IMP	3,776.51 ± 131.08^a^	3,335.62 ± 391.48^b^	3,239.55 ± 211.16^c^
GMP	180.13 ± 26.88^a^	129.50 ± 20.14^b^	101.73 ± 5.37^b^
ATP	93.40 ± 5.58^a^	79.69 ± 12.09^b^	ND
ADP	452.20 ± 105.01	509.28 ± 24.83	532.43 ± 19.77
AMP	115.06 ± 8.82^b^	126.79 ± 2.36^a^	99.32 ± 4.28^c^
Inosine	1,061.78 ± 51.39^c^	1,575.19 ± 82.82^b^	1,828.92 ± 79.36^a^
Hypoxanthine	ND	ND	ND
Total collagen (mg/g, db)	19.59 ± 2.96	20.19 ± 2.48	24.14 ± 6.91
Insoluble collagen (mg/g, db)	8.19 ± 1.42^c^	11.76 ± 1.37^b^	19.25 ± 1.48^a^
Carbonyl content (nmol/mg protein)	3.24 ± 0.51^b^	5.30 ± 1.32^a^	4.58 ± 1.41^ab^

Abbreviations: CB: commercial broiler; KC: Korat hybrid chicken; NC: Thai native chicken; db: dry basis; ND: Not detected.

a-c Mean values within a row with different superscripts differ significantly (*P* < 0.05).

### Collagen Content and Protein Oxidation

Collagen influences the texture and tenderness characteristics of meat. The total collagen content was comparable in all the breeds (*P* > 0.05; Table 2), whereas insoluble collagen was higher in the meat of NC than those of other breeds (*P* < 0.05). Intramuscular collagen is mainly found in the perimysium. No significant difference was observed in the perimysium thickness of the breast muscles between broilers and Thai NC (Koomkrong et al., 2015), which may be the reason for the lack of significant difference in the total collagen content between these 3 chicken breeds. The differences in insoluble collagen content among breeds could be attributed to age. The crosslinks in collagen increase with age, which may have resulted in a higher insoluble collagen content in the breast meat of 16-wk-old NC (Coró et al., 2002). This agreed with previous research (Wattanachant et al., 2004; Intarapichet et al., 2008), which reported that older slow-growing birds exhibited higher collagen crosslinking than fast-growing birds. These results reflect the unique textural properties of each chicken breed.

Protein oxidation in the KC and NC meats appeared to be higher than in CB meat, as evidenced by the protein carbonyl content (*P* < 0.05; Table 2). The variation in protein carbonylation between chicken breeds can be attributed to the different concentrations of myoglobin and the composition of the muscle fiber types. Thai NC contains a higher proportion of oxidative muscle fibers than the muscles of fast-growing chickens, which are more susceptible to oxidation than glycolytic muscle fibers (Jaturasitha et al., 2017; Silva et al., 2018), leading to an increase in protein oxidation in KC and NC meats. Moreover, the lower pH of KC and NC meats (Table 1) indicates a higher H^+^ concentration, which would favor the redox cycle of myoglobin and, hence, its pro-oxidation (Estévez, 2015). The higher protein oxidation in the meats of KC and NC, birds that were older than CB, also demonstrated the effect of age on ROS generation, leading to a greater extent of oxidized muscle proteins. Our study indicated that protein oxidation of raw KC and NC meats occurred to a greater extent than that of raw CB (Cui et al., 2012; Del Vesco et al., 2017). The higher protein oxidation may be responsible for the deterioration in meat quality, such as an increase in toughness and a loss of nutritive value (Lund et al., 2011; Sante-Lhoutellier et al., 2007).

### Vibrational Spectroscopic and PCA Analysis

**Figure 2:**
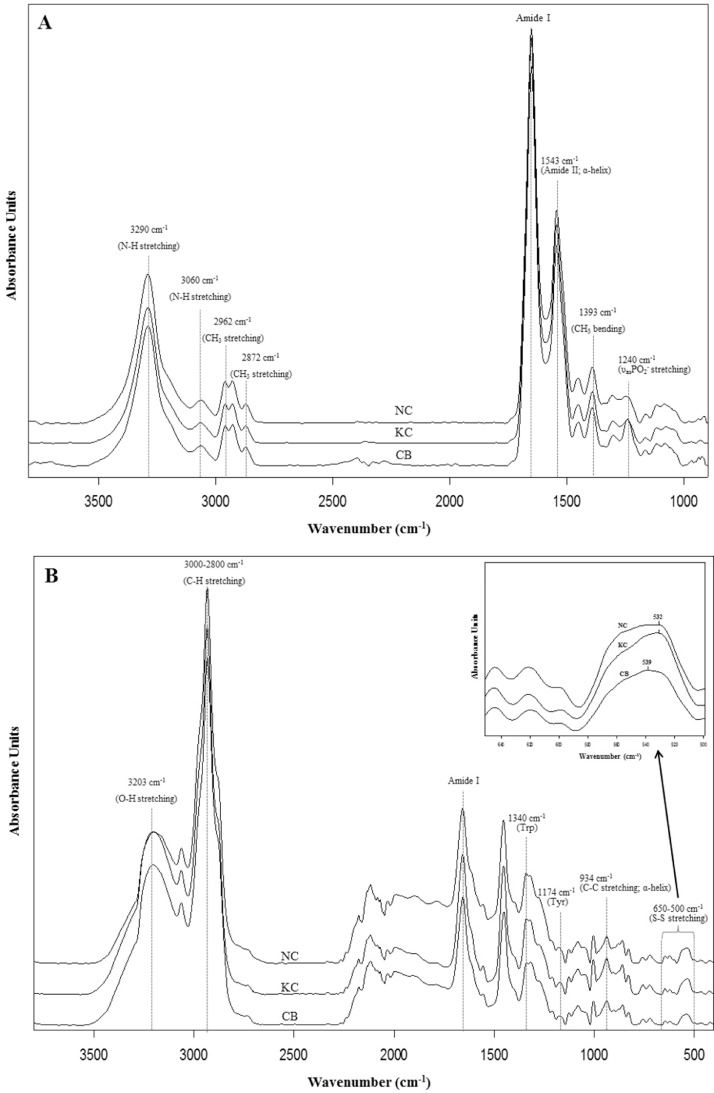
Averaged SR-FTIR (A) and FT-Raman (B) spectra of breast meat from three chicken breeds. Abbreviations: CB, Commercial broiler; KC, Korat crossbred chicken; NC, Thai native chicken.

The representative SR-FTIR and FT-Raman spectra of meat samples are presented in Figure 2A and B, respectively. The detailed band assignments of the IR and Raman spectra, based on the literature, are presented in Table 3 (Li-Chan and Nakai, 1991; Wong et al., 1991; Ngarize et al., 2004; Marinkovic and Chance, 2006; Böcker et al., 2007; Herrero, 2008; Perisic et al., 2011; Lamyaa, 2013; Phongpa-Ngan et al., 2014; Hu et al., 2017; Kang et al., 2017). A significant difference in the SR-FTIR spectra at 1,240 cm^−1^, corresponding to the PO_2_^−^ asymmetric stretching of the phosphodiester groups of nucleic acids (Wong et al., 1991), was observed, with the highest intensity in CB meat (Figure 2A). Moreover, the FT-Raman spectra in the range of 650 to 500 cm^−1^ revealed a distinct band at 532 cm^−1^ in KC and NC meats but at 539 cm^−1^ in CB meat (Figure 2B). These bands corresponded to the S–S stretching of gauche–gauche–trans (*g-g-t*) and trans–gauche–trans (*t-g-t*) conformations, respectively. Thus, the demonstrated S–S bonds of KC and NC meats were in the *g-g-t* conformation, whereas those of CB were in the *t-g-t* conformation, indicating the greater extent of disulfide linkages in slow-growing chickens (KC and NC). The characteristic peaks obtained from the normalized intensities of SR-FTIR and FT-Raman bands of meat from the three chicken breeds are presented in Table 4. The highest absorption band of SR-FTIR at 1,240 cm^−1^in CB meat was consistent with the high content taste-active nucleotides (Table 2). In addition, high intensity of the SR-FTIR spectra at 3,290 cm^−1^ assigned to N–H stretching of amide A and O-H stretching and 3,063 cm^−1^ of N–H stretching of amide B observed in NC meat corresponded with its highest protein content (Table 1). These two IR wavenumbers appeared to be protein markers of chicken meat. The SR-FTIR spectra of NC meat showed high-intensity bands at 2,962 and 2,872 cm^−1^, arising from the asymmetric stretching and symmetric stretching of CH_3_ groups, respectively, and a low-intensity band at 1,393 cm^−1^, originating from CH_3_ bending. These results suggested a high content of hydrophobic residues in NC meat. With regard to FT-Raman spectroscopy, CB meat exhibited a high-intensity peak at 3,203 cm^−1^ (O–H stretching of water), which was correlated with the high moisture content (Table 1). The high-intensity band at 934 cm^–1^ (C–C stretching of an α-helix) was correlated with the high WHC in KC meat (Table 1). The spectral regions of 3,128 to 3,071 cm^–1^ and 951 to 876 cm^−1^ have been reported to correlate well with the WHC of pork meat (Pedersen et al., 2003). Furthermore, the Raman spectra of KC and NC meats exhibited high-intensity bands at 1,340 cm^−1^ (Tyr) and 1,174 cm^−1^ (Trp), respectively, indicating a greater content of aromatic hydrophobic residues in the protein structure of KC and NC meats. The Raman spectra revealed that the KC and NC meats were more hydrophobic, with a higher proportion of S–S crosslinks than CB meat.

**Table 3:** Assignment of IR and Raman characteristics bands of meat from three chicken breeds.

Wavenumber (cm^−1^)		
IR	Raman	Vibrational modes
	3,203	O-H stretching
3,290		N-H, O-H stretching
3,063		N-H stretching
	3,000–2,800	C-H stretching
2,962, 2,872		CH_3_ stretching
1,657–1,648	1,658–1,645	Amide I (α-helix)
1,695–1,674, 1,640–1,610	1,680–1,665, 1,640–1,610	Amide I (β-sheet)
1,657-1,642	1,665–1,660	Amide I (random coil)
1,686-1,662	1,690–1,680	Amide I (β-turn)
1,393		CH_3_ bending
	1,340	Tryptophan (Trp)
1,543		Amide II (α-helix)
1,240		Asymmetric PO_2_^−^ stretching (υ_as_PO_2_^−^ )
	1,174	Tyrosine (Tyr)
	934	C-C stretching (α-helix)
	650–500	S-S stretching (disulfide bonds)
	539	*trans-gauche-trans (t-g-t)*
	532	*gauche-gauche-trans (g-g-t)*

**Table 4:** Normalized intensities of SR-FTIR and FT-Raman bands obtained from three chicken breeds (mean ± SD).

	Normalized bands (x10^−2^)		
Bands assignment	CB	KC	NC
SR-FTIR			
N-H/O-H stretching 3,290 cm^−1^	24.61 ± 0.20^b^	23.64 ± 0.06^c^	25.66 ± 0.20^a^
N-H stretching 3,063 cm^−1^	1.81 ± 0.02^b^	1.87 ± 0.04^b^	2.01 ± 0.05^a^
CH_3_ stretching			
2,962 cm^−1^	2.21 ± 0.02^b^	2.36 ± 0.07^a^	2.43 ± 0.04^a^
2,872 cm^−1^	1.36 ± 0.07^ab^	1.29 ± 0.06^b^	1.45 ± 0.04^a^
CH_3_ bending 1,393 cm^−1^	5.91 ± 0.15^a^	5.86 ± 0.15^a^	5.41 ± 0.23^b^
Asymmetric PO_2_^−^ stretching 1,240 cm^−1^	4.75 ± 0.37^a^	2.20 ± 0.21^b^	2.33 ± 0.62^b^
FT-Raman			
O-H stretching 3,203 cm^−1^	0.84 ± 0.02^a^	0.83 ± 0.03^a^	0.65 ± 0.06^b^
Tryptophan 1,340 cm^−1^	0.10 ± 0.01^b^	0.16 ± 0.01^a^	0.13 ± 0.02^a^
Tyrosine 1,174 cm^−1^	0.06 ± 0.01^b^	0.08 ± 0.00^a^	0.07 ± 0.00^ab^
C-C stretching (α-helix) 934 cm^−1^	0.15 ± 0.01^b^	0.21 ± 0.03^a^	0.16 ± 0.01^b^
S-S stretching (disulfide bonds) 539, 532 cm^−1^	0.12 ± 0.01^b^	0.17 ± 0.07^a^	0.16 ± 0.01^a^

Abbreviations: CB, commercial broiler; KC, Korat hybrid chicken; NC, Thai native chicken.

a-b Mean values within a row with different superscripts differ significantly (*P* < 0.05).

The quantitative estimation of the protein secondary structure obtained from the amide I spectral profile revealed that α-helices were a predominant structure in all three chicken breeds (Table 5). The amide I region in the SR-FTIR spectra indicated that the α-helix structure was most prevalent in CB meat, whereas NC meat contained the highest content of β-turns (Table 5). However, the protein secondary structure obtained from the amide I profile of the FT-Raman spectra was comparable. The amide I band appeared to be more intense in the SR-FTIR spectra than in the Raman spectra. Our study demonstrated that inherent protein secondary structures in the meat of different chicken breeds can be identified via SR-FTIR spectroscopy at the cellular level.

**Table 5:** Relative content (%) of protein secondary structures in meat from different chicken breeds obtained from amide I spectral profile of SR-FTIR and FT-Raman spectra (mean ± SD).

	Relative content (%)		
Protein secondary structures	CB	KC	NC
SR-FTIR			
α-helix	45.95 ± 4.45^a^	38.32 ± 1.59^b^	39.13 ± 1.84^b^
β-sheet	26.85 ± 2.40	26.40 ± 1.54	27.52 ± 3.19
Random coil	12.41 ± 2.04	16.22 ± 1.45	12.83 ± 2.39
β-turn	14.79 ± 1.85^b^	19.06 ± 1.75^ab^	20.53 ± 2.35^a^
FT-Raman			
α-helix	44.54 ± 1.41	44.54 ± 1.41	43.53 ± 0.73
β-sheet	27.81 ± 0.51	27.26 ± 0.07	28.01 ± 0.77
Random coil	16.51 ± 0.57	16.21 ± 1.30	18.28 ± 0.51
β-turn	11.50 ± 1.25	11.99 ± 1.18	10.19 ± 0.83

Abbreviations: CB, commercial broiler; KC, Korat hybrid chicken; NC, Thai native chicken.

a-b Mean values within a row with different superscripts differ significantly (*P* < 0.05).

**Figure 3:**
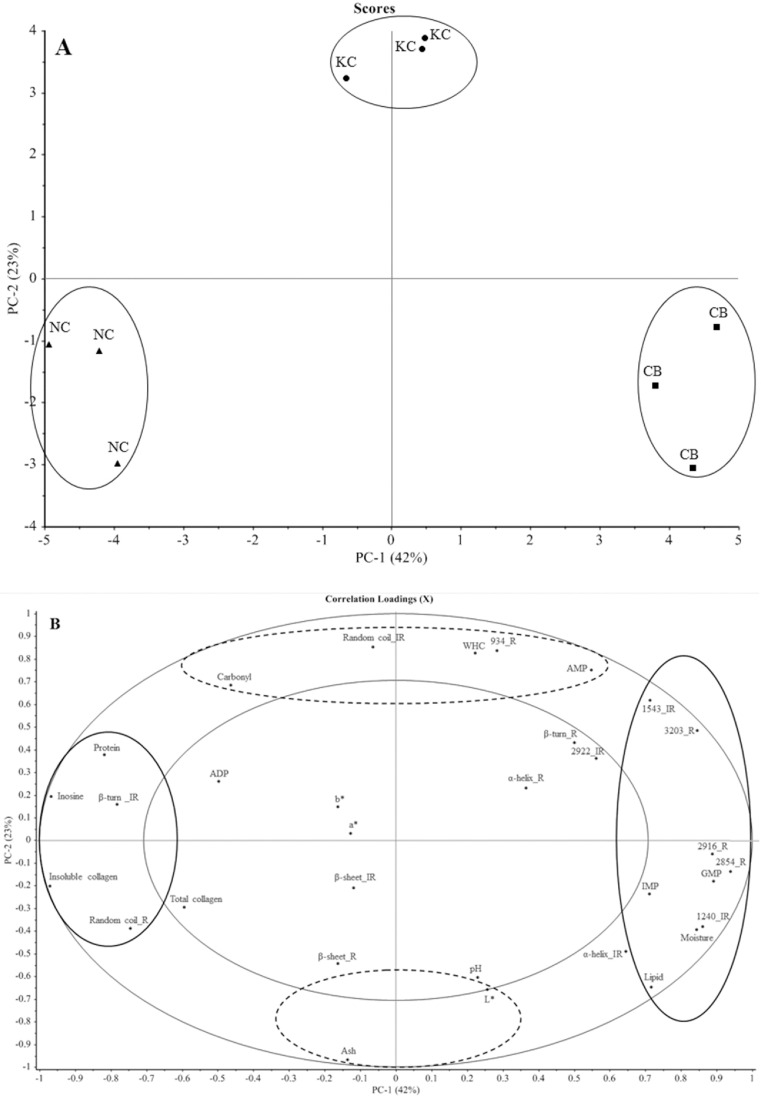
Plot of principle component (PC) scores (A) and correlation loading plot (B) of the averaged SR-FTIR and FT-Raman spectra and quality attributes of breast meat from three chicken breeds. The mean values from each of 3 replicates were used in these three chicken breeds, giving a total of 9 data points. Abbreviations: CB, Commercial broiler; KC, Korat crossbred chicken; NC, Thai native chicken; IR, SR-FTIR spectra; R, FT-Raman spectra; WHC, Water-holding capacity; IMP, Inosine 5′-monophosphate; GMP, guanosine 5′-monophosphate; ADP, adenosine diphosphate; AMP, adenosine monophosphate; L*, lightness; a*, redness; b*, yellowness.

PCA was performed to evaluate the correlation of the SR-FTIR and FT-Raman spectra with the meat quality of these 3 chicken breeds. The plots of the first and second principle component (PC) scores explained approximately 65% of the total variability (Figure 3A). The PCA score plot showed that the 3 chicken breeds were clearly distinguished from each other. Meats from all 3 breeds were clearly separated along PC-1, and KC meat was differentiated from the other 2 breeds along PC-2 (Figure 3A). CB meat was characterized by the high contents of moisture, lipid, and taste-active nucleotides (IMP and GMP) (Figure 3B), which was positively correlated with the O–H stretching (3,203 cm^−1^) and C–H stretching (2,916, 2,854 cm^−1^) of lipids, as measured via FT-Raman spectroscopy. The distinct characteristics of the SR-FTIR spectra of CB also included amide II of the α-helix structure (1,543 cm^−1^) and asymmetric PO_2_^−^ stretching of nucleic acids (1,240 cm^−1^) (Figure 3B). In addition, high-asymmetric PO_2_^−^ stretching of the nucleic acids may be indicative of the high content of taste-active nucleotides in CB meat (Table 2). Contrarily, NC meat aligned in the negative direction of PC-1, which was characterized by high protein, inosine, and insoluble collagen contents, as well as the predominance of β-turns (amide I region; SR-FTIR spectra) and random coil (amide I region; Raman spectra). KC aligned in the positive direction of PC-2, with the distinct characteristics of higher WHC, AMP content, carbonyl content, and random coil structures (amide I region; SR-FTIR spectra), along with a high-intensity Raman band at 934 cm^−1^ (C–C stretching of an α-helix) but low ash and lightness (L*). Our results indicated that SR-FTIR is a powerful technique for monitoring protein conformation in the amide regions, whereas FT-Raman revealed the local environments of protein (tyrosine, tryptophan residues, disulfide bonds, and aliphatic amino acids), moisture, and lipids. Thus, SR-FTIR can be complementary with the FT-Raman technique for the elucidation of chicken meat quality.

## CONCLUSIONS

Chicken meat from CB, KC, and NC birds exhibited distinct physicochemical properties and protein structures. CB meat has high contents of moisture, lipid, and taste-active nucleotides (IMP and GMP). High protein, inosine, and insoluble collagen contents were observed in NC meat. The KC meat had a high WHC, as well as high protein carbonyl and AMP contents. In addition, the predominant secondary structures in NC and KC meats were β-turns and random coils, whereas α-helices were the main secondary structures in CB meat. The intensity of the SR-FTIR peak at 1,240 cm^–1^ was correlated with taste-active nucleotides, and the FT-Raman peaks at 3,203, 2,854, and 934 cm^–1^ were related to moisture, lipid, and WHC, respectively. Vibrational spectroscopy is therefore a potential technique for evaluating quality attributes in chicken meat at the molecular level.
